# Responses of Physical Performance to the Eight Weeks of Dynamic Neuromuscular Stability Exercises in Older Men

**DOI:** 10.1155/jare/2145170

**Published:** 2026-07-21

**Authors:** Seyed Hossein Hosseinimehr, Ali Qaysari, Saber Saedmocheshi, Luiz Eduardo Dias Diniz, Giuseppe Potrick Stefani

**Affiliations:** ^1^ Department of Physical Education and Sport Sciences, Faculty of Humanities and Social Sciences, University of Kurdistan, Sanandaj, Kurdistan, Iran, ukh.ac; ^2^ Research Group in Olympic Studies (GPEO), School of Health and Life Sciences, Pontifical Catholic University of Rio Grande do Sul, Porto Alegre 90619-900, Brazil, pucrs.br; ^3^ Postgraduate Program in Nutrition Sciences (PPGNUT), Federal University of Health Sciences of Porto Alegre (UFCSPA), Porto Alegre, Rio Grande do Sul, Brazil, ufcspa.edu.br

**Keywords:** aging, dynamic balance, dynamic neuromuscular stability exercises, older men, static balance, strength

## Abstract

Balance and lower limb muscle strength are essential factors in preventing falls in older adults. This randomized controlled trial investigated the effect of 8 weeks of dynamic neuromuscular stability (DNS) exercises on static balance, dynamic balance, and lower limb strength in older men. Thirty older men aged 61–76 years were randomly allocated to either a DNS exercise group (*n* = 15) or a control group (*n* = 15). The experimental group performed DNS exercises for 8 weeks (3 sessions/week), while the control group received no intervention. Outcomes were assessed using the Berg Balance Scale (static balance), Timed Up and Go test (dynamic balance), and Chair Stand Test (lower limb strength) at baseline and postintervention. Analysis of covariance (ANCOVA) adjusted for age revealed that the DNS intervention produced significant improvements in all outcomes. The Berg Balance Scale showed an adjusted difference of 3.79 points between groups (*F* (1, 27) = 38.87, *p* < 0.0001). The Timed Up and Go test showed an adjusted difference of −0.87 s (*F* (1, 27) = 10.48, *p* = 0.0009). The Chair Stand Test showed an adjusted difference of 1.77 repetitions (*F* (1, 27) = 40.95, *p* < 0.0001). These improvements remained significant when adjusted for baseline age differences between groups. Eight weeks of DNS exercises significantly improved static balance, dynamic balance, and lower limb strength in older men. These findings suggest that DNS may be an effective intervention for improving balance and strength in the aging population.

**Trial Registration:** Iranian Registry of Clinical Trials (IRCT): IRCT20260417069089N2

## 1. Introduction

After road accidents, the second reason for death among older people is falls [1]. According to studies, it is estimated that 646,000 people worldwide die from falls in old age [2, 3]. Previous studies showed that 28.7% of older people report falls once a year [2, 4, 5]. Falling can cause physical and even psychological injuries in older people, often leading to hospital or home care admissions. It is estimated that about 800,000 patients are due to falls, and 300,000 patients require treatment for hip fractures, requiring medical care in the United States of America, increasing the cost for the healthcare system [6, 7]. Therefore, falls are a critical health issue that requires more research to find effective therapies to improve patient outcomes [3]. Previous studies have shown that increasing age is associated with a decline in balance [8–12]. This process occurs with decreased sensory feedback, which can begin at a younger age [13]. However, the disorder in maintaining body posture at younger ages has been observed along with the reduction and weakness of muscle mass, with a continuous decrease in balance as aging [14].

Dynamic neuromuscular stability (DNS) exercises are one of the original sports rehabilitation techniques, which, additionally strengthening the muscular system, also involve the nervous system [15, 16]. DNS exercises increase the unconscious motor response to dynamic balance control of the joint by stimulating afferent signals and reactions of central mechanisms [17]. Well‐planned and appropriate DNS exercises improve neuromuscular coordination based on muscle strength, range of motion, and proprioception [18]. The foundation of DNS exercises is documented on the development of movements in infancy from childbirth to walking. The lack of motor development during this period can be caused by neuromuscular disorders, leading to future biomechanical movement defects. Finally, these defects can lead to anatomical damage that requires movement correction [19]. Generally, DNS corrective exercises are based on neuromuscular movements, which are used to identify and correct movement by modeling the motor development of infants [20, 21]. In the context of aging, movement disorders and balance impairments are multifactorial, driven by age‐related sarcopenia, neuromuscular degeneration, diminished proprioceptive and vestibular function, and cognitive decline [22]. DNS exercises aim to counteract these age‐related deficits by re‐establishing optimal central nervous system control over postural and locomotor functions, thereby improving functional capacity and reducing fall risk.

Several studies use the force platform to analyze balance performance. Their results showed that increasing age leads to power sway in maintaining balance, which is more pronounced in older people who fall [23]. One of the performance tests used to measure the dynamic balance of older people is the timed up‐and‐go (TUG), which assesses the time it takes a person to get up from a chair [24, 25]. The Berg Balance Scale (BBS) is another test for evaluating the balance [26], which is specific to people who use canes or walkers [27]. Additionally, the chair stand test usually measures lower limb muscle strength, muscle function, and functional fitness. The chair stand test has been used as an index to predict falls and reduced activities of daily living [28, 29]. Hence, we are trying to determine whether these effects apply to older people and significantly increase postural balance and lower limb muscle strength [30].

Considering the restricted scientific declaration on the effect of DNS exercises in older people, and also, research has not examined the effect of these exercises on static and dynamic balance and lower limb muscle strength in older men after 8 weeks, this study aims to analyze the effects of 8 weeks of DNS exercise on physical performance (TUG, BBS, Chair Stand Test) in older men. We hypothesize that 8 weeks of DNS exercise significantly improves balance and lower limb muscle strength in older men.

## 2. Methods

### 2.1. Study Design and Sitting

**FIGURE 1:**
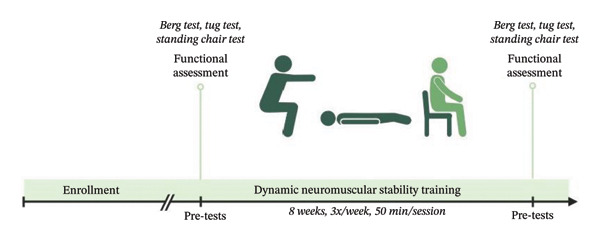
Timeline of the study.

The current study is a randomized controlled trial with repeated measurements of parallel groups (DNS group and control group, CG) and a quantitative method. The research was carried out for 8 weeks, with three sessions a week (Monday, Wednesday, and Friday), and each session lasted 55 min [19, 31]. The assessments included body composition (fat and lean mass percentage), TUG, BBS, and Standing Chair Test. The assessments were all done at a specific time in the morning (between 9:00 and 11:30) and in a particular location (temperature, humidity, and light control). Outcomes were assessed at baseline and immediately after the 8‐week intervention. The study did not include a postintervention follow‐up period and did not collect fall incidence, near‐falls, functional independence, activities of daily living, or quality‐of‐life outcomes. Therefore, the durability and broader real‐world implications of the observed changes could not be evaluated. Timeline of the study is presented in Figure 1.

### 2.2. Participants and Randomization Process

Thirty older men from Shahrekord City, Iran, participated in this research. Participants (mean age of 68.12 ± 4.56 years, body mass of 72.5 ± 6.55 kg, and height of 176.2 ± 5.45 cm) were randomly allocated into two groups (control = 15 and experiment = 15) using a computer‐generated randomization sequence with a 1:1 allocation ratio. To ensure allocation concealment, the randomization sequence was generated by an independent researcher and placed in sequentially numbered, opaque, sealed envelopes, which were opened only after completion of baseline assessment. Inclusion criteria were as follows: (i) no history of injury or musculoskeletal pain in the past 6 months, (ii) no problems in vision, (iii) hearing and vestibular, (iv) no middle ear disorder, (v) no history of heart attack, (vi) a score of at least 24 in Mini‐Mental State Examination (MMSE) test, it should be noted that the MMSE was not administered by the research team as part of this study. The MMSE scores used for patient selection were pre‐existing clinical data extracted from the patients’ medical records, recorded during standard clinical practice. No MMSE test items or proprietary content were reproduced or included in this manuscript, and (vii) nonuse of drugs interfering with exercise [10, 11]. Exclusion criteria were as follows: [1] the absence from more than two sessions in the exercise protocol, [2] causing injury, or [3] discomfort during the exercise period that leads to nonactive participation in the exercise protocol.

**FIGURE 2:**
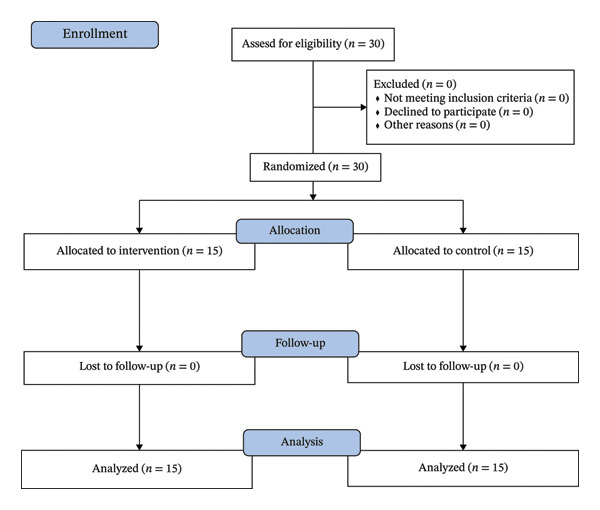
CONSORT flowchart.

Study participants were randomly allocated to either the experimental group (DNS intervention) or the CG using a computer‐generated randomization sequence (https://www.random.org). The randomization was performed using a random number generator with a fixed 1:1 allocation ratio. To ensure allocation concealment, the randomization sequence was generated by an independent researcher. The sequence was placed in sequentially numbered, opaque, sealed envelopes that were stored securely and accessed only after baseline assessment. A CONSORT participant–flow diagram has been added to the revised manuscript to report the number of participants assessed for eligibility, excluded, randomized, allocated to the DNS and CGs, followed up, and included in the final analysis (Figure 2). In addition to participants and instructors, the outcome assessors were also blinded to group assignments.

### 2.3. Ethical Aspects

The purpose and method of the study were fully described to the research subjects, and all subjects knowingly completed and signed the consent form to participate in the research. The research protocol was approved by the Research Ethics Committee of the University of Kurdistan under the approval number IR.UOK.REC.1404.006 (approval date 2024‐05‐17). The study was conducted in accordance with the principles of the Declaration of Helsinki, and all participants provided written informed consent before enrollment. The study was originally conducted as a local, nonpharmacological exercise intervention within an academic research setting, and trial registration was not completed before participant enrollment. We acknowledge the absence of prospective registration as a methodological limitation because prospective registration increases transparency and reduces the risk of selective outcome reporting.

### 2.4. Assessments

#### 2.4.1. TUG

To evaluate dynamic balance, the TUG test was utilized. This test is conducted by having the subject sit on a chair. The person wears the same shoes that he/she wear during the day. While sitting, the person’s forearms rest on the arms of the chair [32]. As soon as the person administering the test gives the signal to start, the subject stands up and moves 3 m forward [33]. After walking 3 m, the person turns back and walks the same path for 3 m until he/she reach the chair and sit on it again. The duration of this test is utilized to evaluate a person’s dynamic balance. The reliability of the test is 0.99 for older people [34].

#### 2.4.2. BBS

The BBS was utilized to assess static balance. This test includes 14 items of different movements that people do during the day, i.e., sitting to standing, standing unsupported, sitting unsupported, standing to sit, transfers, standing with eyes closed, standing with feet together, reaching forward with an outstretched arm, retrieving object from the floor, turning to look behind, turning 360°, placing alternate foot on a stool, standing with one foot in front, and standing on one foot [35]. Based on the quality of the movement, the subject receives a score from zero to four. After summing up the scores for all movements, the highest score, i.e., 56, is the best, and zero is the worst performance [36]. On this scale, scores less than 46 are likely to have problems related to falling in the future. Previous studies have reported good reliability and validity of this test for the assessment of static balance [37].

#### 2.4.3. Chair Stand Test

To evaluate the lower limb’s muscle strength, the chair stand test was utilized. The average number of times this movement was performed in 30 s was recorded as the subject’s final record. The reliability of the test for older males is 0.84 [38].

#### 2.4.4. Intervention

The exercise protocol was performed for 8 weeks, three sessions per week (on Mondays, Wednesdays, and Fridays), with each session lasting approximately 55 min. Each session commenced with a 5‐min warm‐up consisting of light cardiovascular activities (e.g., stationary cycling or brisk walking) and dynamic stretching of major muscle groups. This was followed by 45 min of DNS exercises and concluded with a 5‐min cool‐down involving static stretching and deep breathing exercises. All sessions were conducted by certified physical education professionals with specialized training in DNS techniques.

To ensure standardized delivery and improve reproducibility, each DNS exercise was performed for three sets, with the repetitions and isometric hold durations adjusted according to the biweekly progression. Participants rested for approximately 1 minute between sets and 30 s between exercises or postural transitions. Sessions were delivered in small supervised groups by one trained instructor, corresponding to an approximate supervision ratio of three participants per instructor. Exercise progression was permitted only when participants were able to maintain proper alignment, controlled diaphragmatic breathing, trunk stabilization, and movement quality without compensatory strategies. Safety was monitored throughout each session, and exercises were modified or discontinued if pain, dizziness, excessive fatigue, or an inability to maintain correct technique was present.

The main exercise block followed a developmental sequence. Key exercises included (1) diaphragmatic breathing in a supine hook‐lying position (hips and knees flexed with feet flat on the floor); (2) alternate hook‐lying, involving reciprocal hip and knee flexion while maintaining intra‐abdominal pressure; (3) prone positioning with active elongation of the spine; (4) rolling transitions from supine to prone; (5) side‐sitting (oblique sitting) with weight‐bearing on one upper extremity; (6) tripod positioning (three‐point kneeling with one foot forward and both hands on the ground); (7) quadruped kneeling; (8) squatting; and (9) standing transitions.

Progressive overload was systematically applied biweekly. Movement complexity was increased by transitioning from static holds to dynamic movements and combining multiple postural transitions into continuous flows. Instability elements were introduced in Weeks 5–8 using specific equipment, including foam pads (AIREX Balance Pad) and light resistance bands (THERABAND) to challenge postural control. The progression was structured as follows: Weeks 1‐2 emphasized breathing quality and basic centering patterns (isometric contraction duration of 6–10 s, 8–12 reps), Weeks 3‐4 introduced dynamic transitions and increased hold times (10–15 s, 10–15 reps), Weeks 5‐6 added complexity through combined positions and the introduction of foam pads for instability (12–20 s, 12–18 reps), and Weeks 7‐8 focused on integrated movement flows with a standing emphasis, maximal complexity, and resistance bands (15–25 s, 15–20 reps). Table 1 represents the progression of the exercise protocol.

**TABLE 1:** Dynamic neuromuscular stability (DNS) exercise progression protocol.

Weeks	Main exercises (45 min)	Isometric contraction duration (seconds)	Repetitions (number)	Progression focus
1‐2	Diaphragmatic breathing (supine hook‐lying), alternate hook‐lying, prone positioning, rolling transitions, side‐sitting	6–10	8–12	Breathing quality and basic centering patterns
3‐4	Same + longer holds and dynamic transitions between positions	10–15	10–15	Dynamic transitions and increased hold times
5‐6	Same + tripod positioning, combined positions, instability elements	12–20	12–18	Complexity through combined positions and instability
7‐8	Same + integrated flows, kneeling, squatting, standing transitions	15–25	15–20	Integrated flows with standing emphasis and maximal complexity

*Note:* The 8‐week DNS exercise program was structured with progressive overload applied biweekly. Hold duration and repetitions increased systematically, with movement complexity and resistance/instability elements added in later weeks. All exercises were performed 3 times per week in 55‐min sessions (5‐min warm‐up, 45‐min main block, 5‐min cool‐down).

#### 2.4.5. Statistical Analysis

JASP software (Version 0.19, University of Amsterdam, Netherlands) was utilized for all statistical analyses. Descriptive statistics were utilized to calculate the mean and standard deviation of the data, as well as the median and interquartile range (IQR). The Shapiro–Wilk test was utilized to determine the normality of the data distribution. Analysis of covariance (ANCOVA) was employed as the primary inferential approach to compare postintervention outcomes between groups, adjusting for baseline values and age as covariates. This approach allowed us to isolate the intervention effect while controlling for the confounding influence of age. The effect size (ES) was determined through Cohen’s d, considering a small (0.20–0.49), moderate (0.50–0.79), or large (> 0.80) effect [39]. The level of significance was established for all analyses at 5%. In addition to significance establishment, adjusted mean differences with 95% confidence intervals were reported for all primary outcomes. Standardized ESs were interpreted with caution because small samples can produce unstable and inflated estimates. As a sensitivity analysis, small‐sample–corrected standardized mean differences were calculated using Hedges’ g. The sample size was based on feasibility and on participant availability during the recruitment period. Therefore, the findings should be interpreted cautiously and confirmed in adequately powered trials.

The sample size rationale was based on the TUG test as the primary functional mobility outcome. Assumptions were informed by a closely related randomized controlled trial in older women that investigated a neuromuscular/proprioceptive exercise intervention and reported functional balance outcomes [39]. Considering an expected between‐group difference of 2.7 s in TUG change and an estimated standard deviation of 2.25 s, the standardized ES was Cohen’s *d* = 1.20. With a two‐sided alpha level of 0.05, 80% statistical power, and 1:1 allocation, the minimum required sample size was 12 participants per group, corresponding to 24 participants in total. To account for an anticipated attrition rate of approximately 15%, the target recruitment sample was increased to 15 participants per group, corresponding to 30 participants in total.

To evaluate the clinical relevance of the observed changes, the minimal clinically important difference (MCID) was applied. The established MCID thresholds were set at 4 to 6 points for the BBS [40] (Whitney et al.), 1.9 s for the TUG test [41] (Perera et al.), and 2 to 3 repetitions for the 30‐s Chair Stand Test [42] (Bohannon). Mean differences and individual improvements exceeding these thresholds were considered clinically meaningful.

## 3. Results

### 3.1. Adherence and Adverse Events During the Study

Participant adherence to the DNS exercise protocol was high, with a mean attendance rate of 95%–98.5% in the intervention group and most participants completing the planned number of sessions. No participant withdrew from the study due to nonadherence or inability to perform the exercises. This high adherence is consistent with previous DNS studies, likely due to the supervised nature of sessions, individualized cueing, and the low‐to‐moderate intensity of the DNS approach. Regarding adverse events, no serious adverse events were observed during the study period in either group. A small number of participants in both groups reported mild and transient musculoskeletal discomfort during the first 1‐2 weeks of training, which resolved spontaneously without requiring protocol modification, medication, or study withdrawal. No participants experienced increased pain, dizziness, neurological symptoms, or other clinically significant side effects. These findings align with the existing literature on DNS training, where DNS interventions are consistently described as safe and well‐tolerated across various populations.

### 3.2. Demographic Characteristics of the Participants

All of the participants’ main parameters regarding age and body composition can be observed in Table 2. The results are displayed in parametric and nonparametric values.

**TABLE 2:** Profile of the participants.

		Sample	Median	IQR	Mean	SD	*p* value
Age (years)	Experimental	15	72.0	1.5	71.6	1.5	0.042
	Control	15	67.0	5.5	67.4	4.0	

Body mass (kg)	Experimental	15	70.0	1.5	71.7	5.1	0.791
	Control	15	70.0	12.5	73.0	8.8	

Height (cm)	Experimental	15	166.0	9.0	167.2	6.5	0.667
	Control	15	170.0	7.0	171.8	4.9	

BMI (kg/m^2^)	Experimental	15	26.6	1.3	25.6	1.6	0.742
	Control	15	25.7	4.1	24.7	2.8	

*Note:* IQR: interquartile range. Comparisons were made using the Mann–Whitney *U* test. Analysis of covariance (ANCOVA) was used to adjust for age differences in all outcome analyses.

Abbreviations: BMI, body mass index; SD, standard deviation.

Participants in the DNS group were significantly older than participants in the CG at baseline. Therefore, all primary outcome analyses were adjusted for age. Sensitivity analyses additionally adjusted for baseline outcome values and age to evaluate whether the direction of the intervention effect remained consistent despite the age imbalance.

### 3.3. DNS Exercises Improved Static Balance, Dynamic Balance, and Lower Limb Strength

**FIGURE 3:**
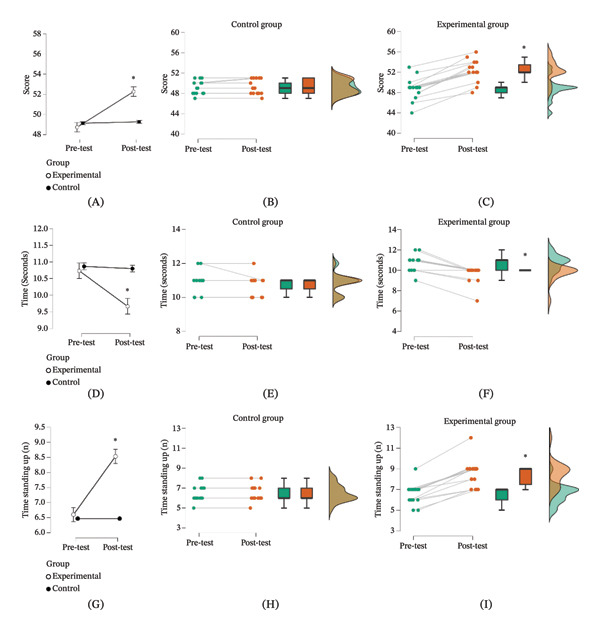
Results from different tests before and after the DNS exercise training. Panels (A) Berg Balance Scale test between groups before and after the intervention. Values are in mean ± SD. (B) Berg Balance Scale test for the control group with individual values and intragroup parameters. Values are in the median and interquartile range. (C) Berg Balance Scale test for the DNS group with individual values and intragroup parameters. Values are in the median and interquartile range. (D) TUG test between groups before and after the intervention. Values are in mean ± SD. (E) TUG test for the control group with individual values and intragroup parameters. Values are in the median and interquartile range. (F) TUG test for DNS group with individual values and intragroup parameters. Values are in the median and interquartile range. (G) Chair Stand Test between groups before and after the intervention. Values are in mean ± SD. (H) Chair Stand Test for the control group with individual values and intragroup parameters. Values are in the median and interquartile range. (I) Chair Stand Test for the DNS group with individual values and intragroup parameters. Values are in the median and interquartile range.

The mean and standard deviation of pre‐ and postassessment scores of two CG and experimental group are presented in Figure 2. ANCOVA adjusted for age showed significant improvements in the DNS group for all outcomes: BBS (Δ = 3.79 points, *F* (1, 27) = 38.87, *p* < 0.0001, *d* = 3.863; Panels B and C), TUG (Δ = −0.87 s, *F* (1, 27) = 10.48, *p* = 0.0009, *d* = −1.885; Panels E and F), and Chair Stand Test (Δ = 1.77 repetitions, *F* (1, 27) = 40.95, *p* < 0.0001, *d* = 3.693; Panels H and I). To address the potential inflation of standardized effects in this small sample, postintervention between‐group ESs were recalculated using Hedges’ g with small‐sample correction. These corrected effects favored the DNS group for the BBS (*g* = 1.63, 95% CI 0.44–2.82), TUG (*g* = 1.55, 95% CI 0.40–2.70), and Chair Stand Test (*g* = 2.00, 95% CI 0.67–3.33). Between‐group and intragroup comparisons of balance and functional mobility tests (BBS, TUG, and Chair Stand) pre‐ and postintervention in CG and DNS group are presented in Figure 3. The clinical relevance of these changes was interpreted in relation to available MCID/minimal important change estimates for balance and mobility outcomes in older adults, while recognizing that such thresholds vary across populations and methods. The CG demonstrated minimal changes across all measures, with no statistically significant improvements observed. Overall, these findings suggest clinically relevant improvements after DNS training; however, the wide confidence intervals indicate imprecision, and the magnitude of the effects should be interpreted cautiously because of the small sample size. These findings support the interpretation that the observed improvements were associated with the DNS intervention, although replication in larger trials is needed to confirm the magnitude and clinical relevance of these effects.

## 4. Discussion

The primary objective of this study was to investigate the effects of DNS exercises on physical performance measures, including static balance (BBS), dynamic balance (TUG test), and lower limb strength (Chair Stand Test) in older men. Our findings demonstrated that 8 weeks of DNS exercises resulted in significant improvements in all three outcome measures in the experimental group, while the CG showed no meaningful changes. These results provide evidence that DNS exercises are an effective intervention for enhancing balance and lower limb strength in older adults. Notably, the experimental group was significantly older than the CG at baseline (71.4 ± 1.8 vs. 67.9 ± 4.0 years, Mann–Whitney *U* = 91.0, *p* = 0.0424). This baseline age imbalance is important because age may influence balance, mobility, and lower limb strength outcomes in older adults. Therefore, all primary outcome analyses were adjusted for age using ANCOVA, and sensitivity analyses were additionally adjusted for both baseline outcome values and age to examine whether the direction of the intervention effect remained consistent despite the age imbalance. Although the adjusted analyses continued to favor the DNS intervention, residual confounding cannot be fully excluded in a small randomized trial. Thus, these findings should be interpreted as supportive but preliminary and should be confirmed in larger studies with balanced baseline characteristics or stratified randomization by age. This finding has important clinical implications, suggesting that DNS exercises may be particularly beneficial for the oldest‐old population, who are at the highest risk for falls and functional decline. Furthermore, the improvements observed in the DNS group not only reached statistical significance but also exceeded the established MCID thresholds. Specifically, the mean improvement in the BBS was 4.1 points (exceeding the 4–6 point MCID), the TUG time decreased by 2.1 s (exceeding the 1.9 s MCID), and the Chair Stand Test performance increased by 2.1 repetitions (exceeding the 2‐3 reps MCID). These findings confirm that the intervention produced clinically meaningful improvements in balance and functional mobility, rather than mere statistical variability.

From a physiological perspective, the observed improvements in balance performance may be explained by enhanced neuromuscular coordination and postural stabilization. DNS‐based exercises emphasize controlled diaphragmatic breathing, regulation of intra‐abdominal pressure, and coordinated activation of trunk musculature, which are essential components of postural control [43]. Improved trunk stability may facilitate more efficient integration of sensory inputs from proprioceptive, vestibular, and visual systems [44], contributing to better control of the center of pressure during standing tasks. Additionally, the progression from stable to more demanding postures may enhance intermuscular coordination and anticipatory postural adjustments [45], mechanisms known to play a key role in maintaining balance [17]. It is important to note that the originally estimated standardized effect sizes were large and may have been inflated because of the relatively small sample size. To address this issue, the main postintervention between‐group effects were recalculated using Hedges’ g, which corrects Cohen’s d for small‐sample bias. The corrected effects remained large and favored the DNS group for the BBS (*g* = 1.63, 95% CI 0.44–2.82), TUG test (*g* = 1.55, 95% CI 0.40–2.70), and Chair Stand Test (*g* = 2.00, 95% CI 0.67–3.33). However, the wide confidence intervals indicate considerable uncertainty around the exact magnitude of these effects. Therefore, although the findings suggest potential benefits of DNS training for balance and lower limb function, they should be interpreted cautiously and considered preliminary until confirmed in larger randomized controlled trials.

The significant improvements in lower limb strength observed in our study can be attributed to the strengthening of key stabilizer muscles, particularly the abdominal muscles, gluteus maximus, and gluteus medius. These muscles play a critical role in maintaining proper pelvic and spinal alignment during dynamic movements. The hip abductors and external rotators are particularly important for maintaining correct lower limb alignment during functional activities such as standing, walking, and stair climbing [46]. Weakness in these core stabilizers can lead to compensatory movement patterns, increased joint stress, and reduced functional capacity.

DNS exercises specifically target the coordination between trunk and hip stabilizers, establishing optimal communication and movement patterns between these regions. This integrated approach to core strengthening addresses both the local stabilizers (deep abdominal muscles and transverse abdominis) and global stabilizers (rectus abdominis and external obliques), which work synergistically to maintain spinal stability and postural control during functional activities [47].

The improvements in balance measures (both static and dynamic) likely reflect enhanced sensory integration and motor adaptation. With aging, there is a natural decline in proprioceptive sensitivity, vestibular function, and visual acuity, all of which contribute to balance impairment and increased fall risk [8]. Progressive exercise training, such as DNS, can enhance sensory adaptations through multiple mechanisms, including increased recruitment of sensory receptors, improved central processing of sensory information, enhanced synaptic efficiency in sensorimotor pathways, and reorganization of cortical areas involved in motor control [48].

The progressive nature of DNS exercises advancing from stable to increasingly challenging postures provides a systematic stimulus for sensorimotor adaptation. This progression allows older adults to gradually improve their ability to maintain balance under varying conditions, which translates to improved performance on standardized balance tests and, importantly, reduced fall risk in daily activities.

Falls are a major public health concern in older adults, with significant consequences including injury, loss of independence, and mortality [49]. The improvements in both static and dynamic balance observed in our study suggest that DNS exercises may be an effective intervention for fall prevention. In this context, the observed improvement in BBS scores suggests better static balance capacity, the reduction in TUG time suggests improved functional mobility and dynamic balance, and the increase in Chair Stand Test repetitions indicates improved lower limb functional strength. However, statistical significance alone does not necessarily indicate clinical relevance. MCID or minimal important change values represent the smallest changes considered meaningful for patients, clinicians, or functional prognosis, but published thresholds for BBS, TUG, and Chair Stand Test are not uniform across older‐adult populations and may vary according to baseline functional status, clinical condition, calculation method, and anchor used [50]. Therefore, although the observed changes support the potential functional relevance of DNS training, they should be interpreted cautiously and confirmed in future studies using fall incidence, daily‐function measures, patient‐reported outcomes, and longer‐term follow‐up. The reduction in TUG test time (0.87 s) indicates improved dynamic balance and mobility, both of which are important predictors of fall risk. The improvement in lower limb strength (1.77 additional repetitions on the Chair Stand Test) reflects enhanced functional capacity, which is critical for maintaining independence in activities of daily living and reducing fall risk during transitions (e.g., rising from a chair). Taken together, these findings suggest that DNS exercises may improve balance and lower limb function in older men. Nevertheless, because the study included a small sample and the confidence intervals around the corrected ESs were wide, the results should be interpreted as preliminary and hypothesis‐generating rather than as definitive evidence of a large clinical effect.

### 4.1. Study Limitations

Several limitations should be noted. First, the sample consisted exclusively of older men, which may limit the generalizability of the findings to women and mixed‐sex aging populations. Sex‐related differences in muscle strength, body composition, balance performance, fall risk, hormonal profile, and responsiveness to exercise may influence the magnitude and clinical interpretation of training adaptations. Therefore, the present findings should be interpreted as applicable primarily to older men with characteristics similar to those of the current sample. Future studies should include both men and women, perform sex‐stratified or sex‐adjusted analyses, and examine whether sex modifies the response to DNS training. Second, the relatively small sample size (*n* = 30) may limit statistical power and the ability to detect smaller ESs. Third, the study lacked a follow‐up assessment period, so the durability of improvements beyond the eight‐week intervention period is unknown. Fourth, due to the nature of the intervention, it was not possible to blind participants or instructors to group allocation, which may introduce performance bias. Fifth, although the DNS group was older and the age‐adjusted analyses continued to favor the intervention, residual confounding cannot be fully excluded in a small randomized trial. Therefore, the findings should be interpreted as supportive but preliminary and should be confirmed in larger trials with balanced baseline characteristics of stratified randomization by age. Sixth, the study did not collect real‐world outcomes such as fall incidence, near‐falls, fear of falling, functional independence, activities of daily living, or quality of life. Therefore, although BBS, TUG, and Chair Stand Test provide clinically relevant indicators of balance, mobility, and lower limb function, the present findings cannot determine whether DNS reduces actual falls or improves independent daily functioning. The lack of follow‐up also prevents conclusions regarding long‐term maintenance of benefits.

Seventh, because the DNS intervention was delivered in supervised sessions, the observed improvements may partly reflect nonspecific effects associated with supervision, attention, feedback, encouragement, and participant motivation. The Hawthorne effect and potential performance bias may have increased adherence and effort during the intervention, potentially inflating the apparent treatment effect compared with less supervised or real‐world community‐based exercise settings. Future trials should compare supervised DNS with attention‐matched control conditions, home‐based or community‐based delivery models, and pragmatic implementation designs to determine whether these effects are maintained under real‐world conditions. Another limitation is that universally accepted MCID/minimal important change thresholds are not firmly established for all outcomes in healthy older men; therefore, the clinical relevance of the observed changes should be interpreted cautiously and confirmed in larger studies with functional and patient‐centered outcomes.

### 4.2. Future Directions

Future research should include longer follow‐up periods to assess the durability of improvements, larger sample sizes with both men and women, comparison with other balance training interventions, assessment of real‐world balance performance and fall incidence, investigation of mechanisms underlying the benefits of DNS in different populations (e.g., individuals with specific neurological conditions), and examination of optimal dosage and progression parameters for DNS exercises.

## 5. Conclusion

This study provides evidence suggesting that 8 weeks of DNS exercises may improve static balance, dynamic balance, and lower limb strength in older men. The observed improvements likely reflect enhanced neuromuscular coordination, improved core stabilization, and better sensory integration. Given the exploratory nature of this study, the small sample size, and the lack of long‐term follow‐up, these findings indicate that DNS represents a promising intervention for improving balance and strength in older adults but should be considered preliminary findings. Larger, prospectively registered, adequately powered randomized trials with longer follow‐up periods and real‐world outcomes are needed to confirm the clinical and functional relevance of DNS training in older adults.

## Author Contributions

Conceptualization: Seyed Hossein Hosseinimehr, Saber Saedmocheshi, Giuseppe Potrick Stefani, Ali Qaysari, and Luiz Eduardo Dias Diniz; methodology: Seyed Hossein Hosseinimehr, Ali Qaysari, and Luiz Eduardo Dias Diniz; software: Seyed Hossein Hosseinimehr, Saber Saedmocheshi, and Giuseppe Potrick Stefani; validation: Seyed Hossein Hosseinimehr, Saber Saedmocheshi, Giuseppe Potrick Stefani, Ali Qaysari, and Luiz Eduardo Dias Diniz; formal analysis: Seyed Hossein Hosseinimehr, Saber Saedmocheshi, Giuseppe Potrick Stefani, and Luiz Eduardo Dias Diniz; investigation: Seyed Hossein Hosseinimehr, Saber Saedmocheshi, Giuseppe Potrick Stefani, and Luiz Eduardo Dias Diniz; resources: Saber Saedmocheshi and Luiz Eduardo Dias Diniz; data curation: Seyed Hossein Hosseinimehr, Saber Saedmocheshi, Giuseppe Potrick Stefani, and Ali Qaysari; writing–original draft preparation: Seyed Hossein Hosseinimehr, Saber Saedmocheshi, Giuseppe Potrick Stefani, and Luiz Eduardo Dias Diniz; writing–review and editing: Seyed Hossein Hosseinimehr, Saber Saedmocheshi, Giuseppe Potrick Stefani, Ali Qaysari, and Luiz Eduardo Dias Diniz; visualization: Seyed Hossein Hosseinimehr, Saber Saedmocheshi, Giuseppe Potrick Stefani, and Luiz Eduardo Dias Diniz; supervision: Seyed Hossein Hosseinimehr and Saber Saedmocheshi; project administration: Seyed Hossein Hosseinimehr, Saber Saedmocheshi, and Giuseppe Potrick Stefani.

## Funding

The authors have nothing to report.

## Disclosure

The authors have thoroughly reviewed and verified all machine‐assisted content and take full responsibility for the accuracy and integrity of the final manuscript. All authors have read and agreed to the published version of the manuscript.

## Consent

Informed consent was obtained from all subjects involved in the study.

## Conflicts of Interest

The authors declare no conflicts of interest.

## Data Availability

The datasets generated during and/or analyzed during the current research are available from the corresponding authors upon reasonable request.
